# Modeling central metabolism and energy biosynthesis across microbial life

**DOI:** 10.1186/s12864-016-2887-8

**Published:** 2016-08-08

**Authors:** Janaka N. Edirisinghe, Pamela Weisenhorn, Neal Conrad, Fangfang Xia, Ross Overbeek, Rick L. Stevens, Christopher S. Henry

**Affiliations:** 1 Mathematics and Computer Science Department, Argonne National Laboratory, S. Cass Avenue, Argonne, IL 60439 USA; 2 Computer Science Department and Computation Institute, University of Chicago, 5640, South Ellis Avenue, Chicago, IL 60637 USA

## Abstract

**Background:**

Automatically generated bacterial metabolic models, and even some curated models, lack accuracy in predicting energy yields due to poor representation of key pathways in energy biosynthesis and the electron transport chain (ETC). Further compounding the problem, complex interlinking pathways in genome-scale metabolic models, and the need for extensive gapfilling to support complex biomass reactions, often results in predicting unrealistic yields or unrealistic physiological flux profiles.

**Results:**

To overcome this challenge, we developed methods and tools (http://coremodels.mcs.anl.gov) to build high quality core metabolic models (CMM) representing accurate energy biosynthesis based on a well studied, phylogenetically diverse set of model organisms. We compare these models to explore the variability of core pathways across all microbial life, and by analyzing the ability of our core models to synthesize ATP and essential biomass precursors, we evaluate the extent to which the core metabolic pathways and functional ETCs are known for all microbes. 6,600 (80 %) of our models were found to have some type of aerobic ETC, whereas 5,100 (62 %) have an anaerobic ETC, and 1,279 (15 %) do not have any ETC. Using our manually curated ETC and energy biosynthesis pathways with no gapfilling at all, we predict accurate ATP yields for nearly 5586 (70 %) of the models under aerobic and anaerobic growth conditions. This study revealed gaps in our knowledge of the central pathways that result in 2,495 (30 %) CMMs being unable to produce ATP under any of the tested conditions. We then established a methodology for the systematic identification and correction of inconsistent annotations using core metabolic models coupled with phylogenetic analysis.

**Conclusions:**

We predict accurate energy yields based on our improved annotations in energy biosynthesis pathways and the implementation of diverse ETC reactions across the microbial tree of life. We highlighted missing annotations that were essential to energy biosynthesis in our models. We examine the diversity of these pathways across all microbial life and enable the scientific community to explore the analyses generated from this large-scale analysis of over 8000 microbial genomes.

**Electronic supplementary material:**

The online version of this article (doi:10.1186/s12864-016-2887-8) contains supplementary material, which is available to authorized users.

## Background

One of the most important elements of an organism’s biochemistry is its ability to produce energy in the form of ATP from nutrients in the environment under a wide variety of environmental conditions. Energy production pathways are of fundamental importance because these pathways define much of the behavior of a microbe and have the greatest impact on the quantitative prediction of biomass and metabolite production yields [1]. Cellular energy generation in microbes is a crucial aspect of metabolic modeling, which depends on environmental factors such as carbon source, electron donor, fermentation capability, presence of electron acceptors, and variations in the electron transport chain (ETC).

Metabolic models provide a valuable means for simulating and understanding energy metabolism based on annotated genome sequences [2]. Recently, tools such as the Model SEED [3–5] have emerged to automate the generation of draft metabolic models to keep pace with the ever growing set of sequenced genomes. However, automatically reconstructed models, and even some curated models, struggle to represent energy biosynthesis accurately primarily for three reasons: (1) genome-scale models integrate complex interweaving pathways that, when under-constrained, can interact to form routes for energy production that are not biologically meaningful or even physically feasible; (2) poor representation of complex and diverse bacterial ETCs and the key pathways related to energy production; and (3) these models often require extensive gapfilling [6] that can lead to the inclusion of some pathways that are not actually present in the species being modeled.

**Fig. 1:**
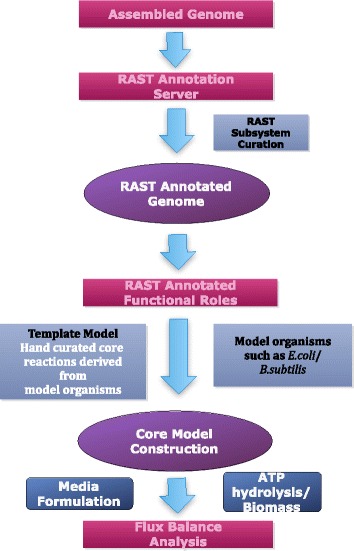
Core metabolic model construction pipeline. The pipeline starts with gene annotations provided by RAST annotation pipeline of assembled microbial genomes. Next, the CMMs are constructed based on a manually curated CMT that consists of biochemical reactions derived from phylogenetically diverse set of model organisms including *Escherichia coli*, *Bacillus. subtilis, Pseudomonas aeroginosa, Clostridium acetobutylicum,* and *Paracococcus denitrificans*. In the final step, FBA is performed optimizing the biomass or ATP hydrolysis as the objective function

Here we present a set of tools and analyses aimed at a focused understanding of energy biosynthesis across the prokaryotic tree of life. Building on important early work in metabolic modeling and engineering [7], we define a “core metabolic model” (CMM), which has a reduced scope consisting of well-annotated central metabolism, fermentation, and ETC pathways. We developed a new high-throughput pipeline for the reconstruction, comparison, and analysis of CMMs for prokaryotic genomes (Fig. 1, Additional file 1: Figure S1). Then we applied our pipeline to the reconstruction and analysis of CMMs for over 8,000 (Additional file 2: Table S1) completely sequenced prokaryotic genomes (http://coremodels.mcs.anl.gov). The CMMs produced by our pipeline had minimal need for gapfilling, demonstrating a key value in CMMs as functional models that are as close as possible to raw annotation output, minimizing model-driven conjectures. In tests of the ATP yield on our models, the results show nearly complete agreement with known values for model organisms. Most importantly, comparative analysis of our core models revealed substantial variation in energy biosynthesis strategies and pathway representation, including variations even at short phylogenetic distances. We observe only a small fraction of theoretically possible combinations of these pathways, with both positive and negative correlations in energy biosynthesis pathways, suggesting a limited number of optimal pathway configurations.

## Results and discussion

### Core model reconstructions and patterns in pathway co-occurrence

**Fig. 2:**
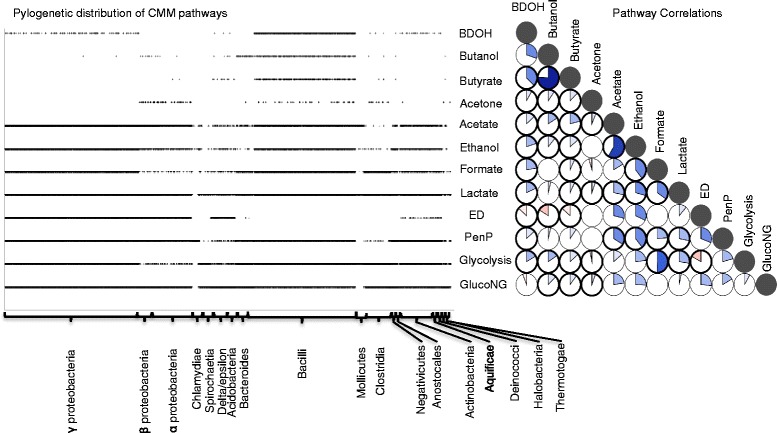
Phylogenetic distribution of CMM pathways and pathway co-occurrence in central metabolism. Presence and absence of 12 key pathways related to energy metabolism including glucose oxidation pathways (glycolysis, ED, TCA cycle, and pentose phosphate) and fermentation pathways (lactate, acetate, formate, ethanol, 2,3-butanediol, butyrate, butanol, and acetone) computed using Boolean rules. Taxonomic groups that are displayed in the horizontal axis of the graph were sorted sequentially as they appear in a 16 s rRNA based phylogenetic tree. The distribution patterns of these key pathways among major phylogenetic groups and pairwise comparisons of pathway presence or absence shows that most pathways are positively correlated. Blue pie slices show comparisons with positive correlations in the clockwise direction while red pie slices show negative relationships in the counterclockwise direction. Relationships shown in pies outlined in bold were consistent across all size classes. Increasing strength of correlation is denoted by increased pie slice size as well as color intensity. Empty pies are relationships that are not significant at *p* < 0.05. Filled pies are self-comparisons

We applied our new core model reconstruction pipeline (Fig. 1; see Methods) to generate 8,179 CMMs belonging to 48 major phylogenetic groups (Additional file 2: Table S2. The number of reactions in our CMMs varied over threefold from 40 to 163 across taxonomic groups (Additional file 1: Figure S2). CMMs were constructed based on a core model template (CMT) that consists of a highly curated set of biochemical reactions derived from a diverse set of model organisms. We selected ~200 unique reactions (Additional file 2: Table S3) that comprise 12 key energy biosynthesis pathways linked to central metabolism (Fig. 2, Additional file 1: Figure S1) and variations of bacterial ETCs (see Methods). These pathways include glucose oxidation pathways and fermentation pathways (Fig. 2, Additional file 1: Figure S1). The presence and absence of each pathway was determined using a set of Boolean rules (Additional file 2: Table S9) based on reactions present in the CMM (Fig. 2, Additional file 1: Figure S1 and Additional file 2: Table S4).

Although the pathways included in CMMs are fundamental to energy generation, not all pathways are present in every genome. Individual pathways were annotated as present in as few as 106 (e.g. acetone fermentation) and as many as 6,694 (e.g. lactate fermentation) genomes. We examined pairwise relationships among all pathways present in CMMs in order to understand variation in core metabolism across this diverse set of microorganisms. In this analysis, we filtered out 4062 CMMs from our dataset because their associated genomes were overly close phylogenetically to other CMMs (many mapped to different versions of genomes with the same taxon ID). We found an overall pattern of positive co-occurrence (88 % of co-occurrences were positive; Fig. 2) among pathways suggesting that core metabolism is diverse, yet consists of a set of interdependent sub-modules. Once we controlled this analysis for CMM size (see Methods), we found slightly fewer positive co-occurrences, with small CMMs having 62 % positive co-occurrences, medium CMMs having 71 % positive co-occurrences, and large CMMs having 79 % positive co-occurrences. We found little evidence to support the idea of substrate competition among pathways [8], regardless of CMM size, despite many fermentation pathways deriving from the same substrate. To the contrary, the two strongest positive correlations were between pairs of pathways that branched from a single substrate: the butanol and butyrate pathways (r2 = 0.76; Fig. 2) and the acetate and ethanol pathways (r2 = 0.59; Fig. 2). Fermentation pathways deriving from the same substrate tended to have strong positive relationships among themselves and also tended to respond similarly to the presence or absence of other pathways (Fig. 2).

Seven negative relationships between pathways were identified, with five of these being consistent across CMM size classes. These consistent negative co-occurrences may represent physiological trade-offs between adaptation toward maximizing biomass yield and growth rate. A yield versus growth rate trade-off has been previously suggested [9, 10], and is supported by the multiple negative relationships with the Entner-Doudoroff (ED) pathway observed here. For example, we found a negative correlation between ED and glycolysis. ED is found in a wide range of genomes, despite having a lower ATP production efficiency [9]. Meanwhile, glycolysis is more efficient with twice the ATP yield, but it incurs a greater enzymatic cost [9], potentially leading to slower growth than ED (Fig. 2, Additional file 1: Figure S1). ED also had non-positive relationships with fermentation pathways containing three or more reactions (Fig. 2). These longer fermentation pathways were found primarily in fermentative anaerobes expected to grow under energy-limited conditions, which have been shown to favour energy-efficient glycolysis [9]. In these organisms, continued selection pressure for maximizing ATP production may have led to an overall negative relationship between these fermentation pathways and ED.

### ETC variations, predictions and ATP yield in core models

**Fig. 3:**
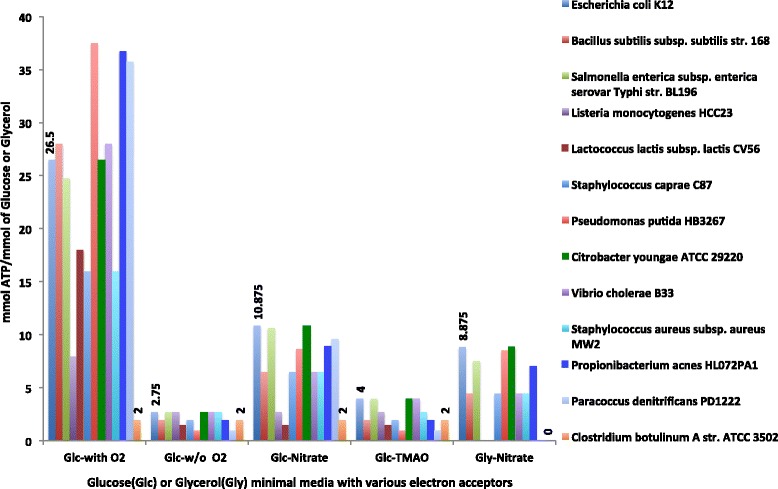
Predictions of ATP yields using FBA on selected core models. The ATP yield predictions were simulated in presence of aerobic, anaerobic electron acceptors (nitrate, TMAO) and without any electron acceptors. Glucose or glycerol was used as the carbon source. Labeled bars show the mmol of ATP/mmol of glucose/glycerol for *Escherichia coli K12* and *Clostridium botulinum* A str. ATCC 3502. ATP hydrolysis is used as the OF for FBA simulations

As metabolic models generally require an objective function (OF) that is optimized during flux balance analysis (FBA) to predict flux profiles, we explored two OFs (see Methods). In order to quantitatively predict energy biosynthesis in CMMs, we used the ATP hydrolysis reaction (ATP + H_2_O - > ADP + Pi + H^+^) as one OF. Using this OF we performed FBA on seven minimal media conditions (Additional file 2: Table S10, http://coremodels.mcs.anl.gov) with a range of electron acceptors to determine the ATP yield under various environmental conditions (Additional file 2: Table S5). These models were not subjected to gapfilling and the predictions were based solely on reactions derived from existing annotations. ATP production depended on the carbon source used, type(s) of electron acceptors available in the media, and the ability to recycle cofactors through the fermentation pathways. This analysis demonstrated a strong capacity for CMMs to capture variations in growth yields and flux profiles based on the electron donor and acceptors present in the media (Fig. 3). When grown in the presence of oxygen, facultative anaerobes, such as *Escherichia coli* and *Pseudomonas putida* preferentially use oxygen as the preferred electron acceptor [11, 12]. Thus aerobic conditions using glucose as the primary carbon source resulted in the highest yields for these organisms, enabled by oxidative phosphorylation activity. FBA simulations also showed variations in ATP production among different carbon sources. For example, when *E.coli* or *Salmonella enterica* were grown anaerobically in glucose or glycerol with nitrate as the electron acceptor, predicted ATP production from glycerol (per-mol basis) was lower than ATP production from glucose (per-mol basis), as expected given that glycerol is a more oxidized carbon source (Fig. 3). We also note that when CMMs are grown aerobically, in some cases the flux distributions show that they use aerobic respiration in combination with fermentation. We notice that about 12 % (716) of the models that are able to produce ATP show this behavior. This result agrees with observed behavior when organisms are grown under laboratory conditions [13–16]. Variations in anaerobic respiration also resulted in differing yields due to differences in the number of protons pumped out of the cell membrane in response to particular electron acceptors and due to differences in the degree of substrate-level phosphorylation. Some obligate anaerobic organisms belonging to the class Clostridia (e.g. *Clostridium acetobutylicum*) have neither aerobic nor anaerobic respiration; hence such organisms use fermentation as the sole means of ATP production [17]. Their yields in our simulations were constant regardless of the electron acceptors present (Fig. 3). Thus, CMMs are accurate enough to capture ATP yields by integrating only relevant ETCs based on consistent RAST annotations. Our E. coli model predicts ATP yields under aerobic respiration and anaerobic fermentation that closely agree with the theoretically determined values described in Kaleta. et al and Muir. et al respectively [18, 19]. Specifically, we predict 26.5 mmol ATP/mmol of glucose during aerobic growth (literature value 26) and 2.75 mmol ATP/mmol of glucose during anaerobic fermentation (literature value range 2.8–3.2). Furthermore, analysis of CMMs shows that organisms such as *Bacillus subtilis* or *Streptomyces coelicolor,* which are classified as obligate aerobes in the public domain, in fact do have the ability to respire anaerobically in the presence of nitrate. These predictions are in agreement with previous studies done on these organisms [20, 21]. Analysis of CMMs can shed light on respiratory capabilities of any sequenced bacterium and generate hypotheses regarding which sets of environmental conditions favour its activity. A complete list of FBA results from our CMM simulations can be found in Additional file 2: Table S5.

Fermentation is an essential process for obligate anaerobes to produce ATP and for many facultative anaerobes, which use it to produce ATP when suitable exogenous electron acceptors are not present. It is also important for metabolic engineering applications, as it is a primary means of producing many biofuel molecules. Analysis of CMMs for the ability to produce fermentation products showed that obligate anaerobes and facultative anaerobes are able to use a variety of fermentation pathways when oxidizing sugars under anaerobic conditions. For example, FBA simulations of a taxonomically diverse set of organisms including facultative anaerobes and anaerobes grown in glucose solely by fermentation, show ATP yields around 2 mmol ATP/mmol of glucose for wide range of bacteria [1, 17] (Fig. 3, Additional file 2: Table S5). In our analysis we found that fermentation pathways, including formation of formate, ethanol, and acetate, are conserved in the classes Bacilli and alpha, gamma and beta preoteobacteria (Additional file 1: Figure S3). 91 % of acetate, 93 % of ethanol and 84 % of formate producing pathways are present in models belonging to one of those four classes. Volatile fermentation products such as acetone, butyrate, butanol, and 2,3 butanediol (BDOH) are conserved mostly in organisms belonging to the Bacilli and Clostridia classes. A complete list of FBA results from these simulations can be found in Additional file 2: Table S5. A complete list of the organisms and the presence and absence of fermentation pathways can be found in Additional file 2: Table S4.

### Coverage of the core model template

Our reconstruction of CMMs for over 8000 microbial genomes provides a means of evaluating the extent to which the annotations and biochemistry comprising our core-model template are sufficient to capture at least one of the energy biosynthesis strategies for each sequenced organism used in this study. To conduct this evaluation, we simulated FBA on all CMMs in seven media conditions using ATP production as the OF. Our analysis shows that about 6,600 (80 %) of the CMMs have some type of aerobic electron transport chain (ETC), whereas about 5,100 (62 %) have an anaerobic ETC; and 1,279 (15 %) of CMMs do not have any ETC. Furthermore, we see that 5,291 models (65 %) were able to produce ATP in glucose minimal media aerobically, while up to 61 % of the CMMs were able to produce ATP with each of the alternate electron acceptors (AEA) examined here. 4,440 (54 %) CMMs were able to grow solely by fermentation when no electron acceptors were present. If AEAs were present in the medium, then CMMs with ETCs tended to use anaerobic ETCs via reduction of the terminal AEAs. This study demonstrates that using CMMs in standard FBA while maximizing energy production can produce accurate predictions for a wide range of organisms.

**Fig. 4:**
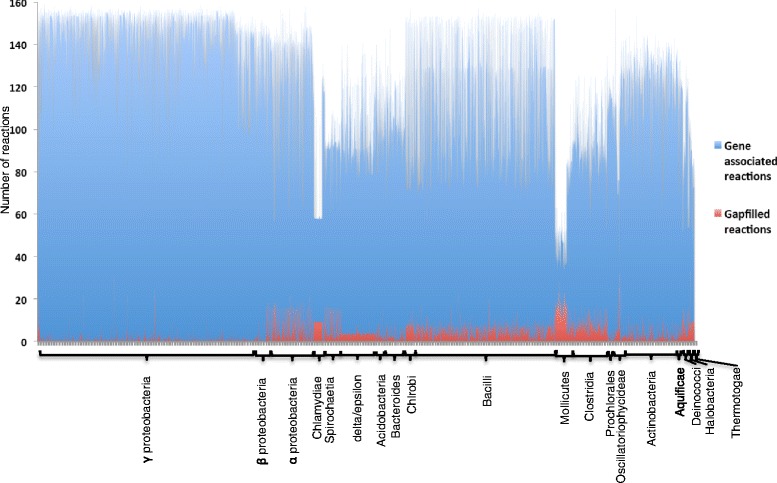
Number of gapfilled reactions that are required in CMMs in order to produce all biomass precursors. Blue bars represent the gene-associated reactions and the red bars represent the gapfilled reactions for all CMMs used in this study. The height of the bars represents the number of reactions. CMMs are grouped according to taxonomical groups

We also identified 2,495 (30 %) CMMs those were unable to produce ATP under any of the tested conditions (Additional file 2: Table S6). We found two explanations for the lack of ATP production in these species: (1) many species are parasitic and have lost key genes in their central metabolism which results these organisms unable to oxidize glucose or glycerol to produce energy and biomass, and (2) annotation inconsistencies or missing annotations in the respective genomes. To explore the extent of key missing reactions in central metabolic pathways, we simulated the CMMs with a biomass OF based on biomass precursor stoichiometry derived by Varma and Palsson (see Methods, Additional file 1: Figure S1 and Additional file 2: Table S12). Models were subjected to gapfilling [6] using glucose as the sole carbon source and oxygen as the electron acceptor. Our analysis shows 3,415 models (42 %) across different phyla did not require any gapfilling to produce all 12 central carbon biomass precursors (Additional file 1: Figure S1). Of the remaining 4,667 models that required gapfilling, 3,183 (66 %) required five or fewer reactions to be added or modified in order to generate all biomass precursors (Fig. 4, Additional file 2: Table S7). It is possible that these models did not grow on glucose because they lack the pathways needed to utilize glucose as a sole carbon source. Thus we explored the capacity of these models to utilize one or more alternative carbon sources, including glycerol, lactate, succinate and ribose. This study revealed that only a small percentage of the models that failed to grow on glucose (<0.03 %) were able to utilize one of these alternate carbon sources instead (Additional file 2: Table S11). From this result, we can conclude that most of the models that fail to grow on glucose do so because of gaps in the biosynthetic pathways for the production of one or more biomass precursor compounds. We applied flux balance analysis with these models to identify which specific biomass precursors could not be produced by each model. This data is provided in Additional file 2: Table S7.

In our gapfilling analysis, we found that over 1000 organisms were missing an annotation for the anaplerotic reaction “Pyruvate carboxylase (EC 6.4.1.1)” that is required to supplement oxaloacetate during glucose oxidation in Gram positive bacteria [17]. Among heavily gapfilled models, many of the organisms that were identified belonged to genera known for either parasitic or pathogenic lifestyles including *Streptococcus, Clostridium, Lactobacillus, Bifidobacterium, Enterococcus, Helicobacter*, and *Campylobacter* as well as more apparently parasitic bacteria with small genomes in the genera *Rickettsia, Ureaplasma, Borrelia, Chlamydia, Treponema* and *Mycoplasma* [22, 23] (Additional file 2: Table S7). These organisms lack the ability to synthesise a range of intermediates in central metabolic pathways, primarily in the TCA cycle, which prevent their growth in glucose. In addition, this analysis was able to capture the reactions and metabolites that are absent in these organisms due to a loss of genes in glycolysis, pentose phosphate pathway, and the TCA cycle, which are the key precursors for synthesis of amino acids, vitamins, cofactors and lipids [1] (Additional file 1: Figure S1). We suggest that this type of analysis is useful in screening likely parasitic organisms. A complete list of organisms and their gapfilling analysis is available in Additional file 2: Table S7.

### Exploration and analysis of CMM pathways in a phylogenetic context

We explored the potential presence of annotation inconsistencies in our models by examining CMM biochemical pathways within a phylogenetic context (Fig. 2, Additional file 1: Figure S4). We found that missing or incorrect annotations can be systematically recognized and fixed with high accuracy when genome annotations are analysed in the context of biochemical pathways. Having the ability to visualize these pathways with respect to phylogenetic relationships (Additional file 1: Figures S4 and S5), annotators can see the propagation of incorrectly annotated pathways in closely related genomes in order to correct them. We also demonstrate the value of focusing on the most well curated pathways first, as with these pathways errors may be more easily separated from novel biology.

In our analysis we identified multiple missing or incorrect annotations including transporter genes, key genes in central metabolism and fermentation pathways. For instance, through our analysis we noticed an aerobic ETC present in obligate anaerobes belonging to the genus *Bacteroides*. This error was due to an incorrectly annotated “ubiquinol cytochrome oxidase” gene throughout this genus. Usually ubiquinol-based cytochrome oxidases could not be found in obligate anaerobes. We corrected this issue in our models. Additionally, we improved the specificity of our annotations where appropriate, such as “cytochrome O ubiquinol oxidase subunit IV (EC 1.10.3.-)” instead of “ubiquinol cytochrome oxidase”. We then integrated these new more-specific functions into our template. We also noticed that the genus *Acinetobacter* does not appear to have functional glycolysis or ED pathways for the degradation of glucose, yet previous studies have suggested that many *Acinetobacter* species are able to utilize the ED pathway for oxidization of glucose [24, 25]. We found that all enzymes in the ED pathway are consistently annotated in *Acinetobacter* except for the enzyme “Gluconolactonase 3.1.1.17”. This apparent inconsistency may in fact be biologically accurate, because the reaction catalysed by this enzyme has been shown to occur spontaneously, and thus the enzyme may not be necessary in every organism containing the broader pathway [26].

It has been known that some members of *Streptococcus* produce formate during fermentation [27], yet no annotations representing formate transporters were found within the *Streptococcus* genus. We also identified key missing gene-protein reaction associations within the CMM template, such as Polyphosphate glucokinase (EC 2.7.1.63), a gene that is abundant in many Actinobacteria [28]. Annotation inconsistencies can be a result of multiple factors including poorly sequenced areas of the genomes, assembly errors, and missed or incorrectly assigned annotations. Through this study we also determined that the genome annotations associated with important reducing reactions for iron, chromium, sulphur, and sulphur derivatives are either not present or not fully propagated among the RAST annotated genomes. In RAST, the consistent propagation of functional annotations is driven by the creation, curation, and maintenance of annotation subsystems [29]. Often, when a subsystem is missing, this is due to a lack of literature data required to accurately assign constituent functions across a diverse set of microbial genera. Proper annotation of these reducing reactions will ultimately permit the prediction of at least one energy production strategy for additional organisms, further improving the coverage of energy production by our CMMs. The approach we used in this study to explore and identify inconsistent or missing annotations in our CMMs by evaluating the coverage of our core model template, comparative analysis of complete biochemical pathways across the microbial tree of life, and gapfilling analysis all represent promising routes to producing consistent annotations.

## Conclusions

Here we present CMMs and comparative analysis for over 8,000 completely sequenced genomes in diverse phylogenetic groups that are derived from a manually curated core model template. Unlike the complexity of genome scale models, CMMs are simpler, offering a quick and accurate way of determining: (i) respiration type(s) (Additional file 1: Figure S5 and Additional file 2: Table S8) and ATP yield predictions (Fig. 3), (ii) electron acceptors that can be reduced during anaerobic respiration (Additional file 2: Table S5), (iii) ability to produce useful fermentation products (Additional file 1: Figure S3 and Additional file 2: Table S4), (iv) presence/absence of functional pathways in central metabolism (Additional file 1: Figure S4 and Additional file 2: Table S4) and (v) evaluate ability to produce key pathway intermediates in central metabolism which are precursors of essential biomass compounds (Additional file 2: Table S7). Having integrated a set of highly curated reactions that represent ETCs, fermentation, and central metabolic pathways, CMMs are able to predict ATP yield variations under aerobic and anaerobic conditions mediated by anaerobic electron acceptors present in the growth medium (Fig. 3) [18, 30]. Using glucose or glycerol as the sole carbon source, we found ~30 % (2,495) of the genomes (Additional file 2: Table S6) used in this study could not be simulated to produce ATP solely based on existing RAST annotations due to incomplete or missing annotations that mapped to reactions comprising the energy producing pathways and ETCs. A major piece of missing ETCs consisted of key reducing reactions for inorganic electron acceptors including iron, chromium, and sulphur that are not consistently annotated across the prokaryotic tree of life. Annotation inconsistencies and missing annotations identified in this study evaluate the quality of RAST annotations and highlight the areas where more attention is needed. Metabolic pathway determination data (Additional file 2: Table S4) and respiration type determinations (Additional file 2: Table S8) are a valuable resource in recognizing inconsistent annotations across the tree of life, even at short phylogenetic distances. Using pathway determinations we identified patterns in pathway co-occurrence and identified potential physiological trade-offs that may influence the ability of organisms to maintain individual central metabolic pathways (Fig. 2). While these tools and analyses were developed specifically to examine central metabolism, the approach is easily scalable to consider the entire metabolic network or other subsets of metabolism of interest enabling researches to address specific research goals.

## Methods

### Core model reconstruction pipeline

CMMs were built on the previously developed metabolic modeling tool Model SEED [3], where the model construction pipeline begins with gene annotations of microbial genomes provided by RAST [29]. This combined use of RAST annotation and Model SEED reconstruction results in high-quality genome annotations, enzyme identification, reaction network assembly, and thermodynamic analysis of reaction reversibility. Our reconstructions of CMMs were achieved by creating a “core model template” (CMT), which consists of a highly curated set of biochemical reactions derived from a well-studied, phylogenetically diverse set of model organisms including *E. coli*, *B. subtilis, Pseudomonas aeroginosa, Clostridium acetobutylicum,* and *Paracococcus denitrificans* [1, 17, 31–35].

In total, we selected ~200 unique reactions (Additional file 2: Table S3) comprising glucose oxidation pathways (glycolysis, ED, TCA cycle, and pentose phosphate pathway) and fermentation pathways (producing end products: lactate, acetate, formate, ethanol, 2,3-butanediol, butyrate, butanol, and acetone) linked directly to central carbon metabolism [36] (Additional file 1: Figure S1) as well as variations in bacterial ETCs [37–46]. These pathways were chosen because they are found across a phylogenetically diverse group of organisms and are relatively well studied and consistently annotated in RAST. We excluded some ETC variants known to be important to energy biosynthesis in some organisms (e.g. sulphate reduction pathways) because they were either not broadly distributed or not consistently annotated by RAST. Absence of these variations of ETCs may result in an inflated number of organisms unable to grow in our FBA simulations due to exclusion of these pathways of known importance, resulting in a conservative estimate of CMM coverage.

In the initial step of the pipeline, genome annotations generated by RAST are applied in combination with our CMT to generate a set of gene protein-reaction (GPR) associations used to reconstruct each CMM (Additional file 2: Table S2). This automated reconstruction process is explained in detail in Henry, DeJongh et al. (2010). The paired CMT and GPR associations were then applied to build CMMs for 8,179 genomes using the Model SEED model construction service. This service has recently been made publicly available for users through KBase (www.kbase.us) services, which was built, in part, from RAST and Model SEED. The core metabolic model construction pipeline with supporting commentary can be accessed through KBase Narrative interface at https://narrative.kbase.us/narrative/ws.15253.obj.1. The reconstruction of all models was completed in less than 24 h. It is important to note the flexibility of the model construction process. In this study we designed a CMT supported by RAST annotations, yet additional templates may be constructed based on other annotation databases with their own annotation ontologies. These templates can then be used to construct metabolic models specifically tailored to address unique research goals.

Metabolic models generally require an OF [47] that is optimized during flux balance analysis to predict flux profiles. In our CMMs, we explored two OFs: a biomass biosynthesis objective function and an ATP hydrolysis objective function. While CMMs do not include the amino acids, nucleotides, lipids, and cofactors that are typically included in the biomass biosynthesis objective function of genome-scale models, they do include the central carbon precursor metabolites for these compounds. Thus the biomass biosynthesis OF for our CMMs was constructed based on the biomass precursor stoichiometry derived by Varma and Parlsson [48] and used in one of the earliest models of *E. coli* [48, 49] (Additional file 1: Figure S1). Coefficient values for NADPH and Erythrose-4-Phosphate have been modified in our OF from the original source material (Additional file 2: Table S12). When analysing CMMs using the biomass biosynthesis OF, we found that occasionally gapfilling was required to enable synthesis of all essential biomass precursors (Fig. 4). To permit a focused study of energy biosynthesis in our models without gapfilling, we developed a second OF for our CMMs consisting only of the ATP hydrolysis reaction: ATP + H_2_O - > ADP + Pi + H^+^. Using this OF, we computed ATP production yields in all models without any gapfilling; hence, these computations were based solely on reactions derived from existing RAST annotations.

### Integration of electron transport chains into core models

Many current metabolic models have a simplified version of ETC, lacking representation of multiple steps of proton pumping reactions or lacking reactions that are related to the reduction of anaerobic electron acceptors (e.g. nitrate, dimethyl solfuxide) resulting in inaccurate prediction of ATP production. These issues persist because of difficulties integrating ETCs into models. In designing ETCs for the CMMs, we incorporated well-studied variations of ETCs in model organisms that were supported by consistent RAST annotations, and we integrated these variations into our CMT (Additional file 2: Table S3). This CMT includes new GPR associations for ETCs to facilitate diverse proton pumping reactions, terminal electron acceptor reducing reactions, and key fermentation pathways, which increased the accuracy of the ATP yield predictions (Fig. 3). As more consistent annotations become available representing a broader range of ETC variations, those GPR associations will be added to our template to expand the coverage of ETC by our CMMs. Organisms that contain at least one type of respiration chain are classified as aerobic or anaerobic, while organisms that contain both aerobic and anaerobic respiration chains are classified as facultative (Additional file 1: Figure S5 and Additional file 2: Table S8).

### Pathway determination in central metabolism

One of the key advantages of metabolic models is the ability to predict phenotypes based solely on genomic sequence. We have examined microbial phenotypes through comparative analysis of the presence or absence of 12 key energy biosynthesis-related pathways (Fig. 2) and respiration types (Additional file 1: Figure S2 and Additional file 2: Table S8). We developed a set of Boolean rules to determine the presence and absence of each pathway based on reactions present in the CMM. The Boolean rules allow for alternative reactions within an individual step of each pathway, but every step of each pathway must be annotated in order for the pathway to be classified as present. Next, we organized all CMMs by their taxonomic groups against pathway presence and absence data (Fig. 2). Taxonomic groups that are displayed in the horizontal axis of Fig. 2 were sorted sequentially as they appear in a 16S rRNA based phylogenetic tree (see Generation of Phylogenetic Trees and Pathway Visualization). As a result, we were able to analyse the distribution patterns of these key pathways among major phylogenetic groups.

Pairwise comparisons of the presence or absence of individual pathways were conducted using the Hmisc [50] and corrgram [51] packages in R [52] version 2.15.1 to examine pathway co-occurrence patterns across CMMs. Co-occurrence analysis was performed after the removal of models associated with identical 16S sequences from the dataset; this criterion was applied strictly, with only one representative model selected for each sequence analysed (*n* = 4117). To control for the increased likelihood of positive co-occurrences with larger CMM size, all analysis were performed on three size classes: small (<93 reactions, *n* = 1353), medium (93-133 reactions, *n* = 1416), and large (>133 reactions, *n* = 1348), as well as the entire non-redundant dataset. Boolean vectors containing presence/absence information for each of the 12 key energy biosynthesis pathways was used to examine the diversity of pathway combinations present within the CMMs. Pathway presence and absence data is included for all CMMs in Additional file 2: Table S4.

### Generation of phylogenetic trees and pathway visualization

We constructed a phylogenetic tree for all CMM genomes using the SEED server tools [53]. Specifically, we extracted all copies of 16S ribosomal RNA sequences from the complete list of 8,179 genomes. This was done by blasting these genome contigs against a curated database of 92 diverse 16S references. For each genome, the matched sequence with the best bit score was kept (8 genomes were removed because they did not produce a 16S hit that passed the quality threshold). The remaining sequences were aligned using the MAFFT aligner [54] and a phylogenetic tree was built using the FastTree2 program [55]. The resulting phylogenetic tree was collapsed at an evolutionary distance of 0.01 under the general time-reversible model of nucleotide evolution. That is, for each maximal subtree where all the pairwise leaf distances are below 0.01, the leaf that corresponds to the genome with the highest sequence quality (based on assembly and annotation metrics) in the group was chosen to represent the subtree. This process is akin to clustering genomes into operational taxonomic units (OTU^98.5^) and resulted in a representative tree of 1,864 genomes.

We then mapped presence/absence information of subsets of the key pathways to each genome as a Boolean vector (e.g., “Glycolysis: yes, Gluconeogenesis: no, Entner-Doudoroff: yes” or Aerobic: yes, Anaerobic: yes, Facultative: yes) and drew circular trees using the iTOL tree visualization tool [56]. The tree branches are color coded by the pathway vector, and the leaves are labeled by species names at the outer ring (Additional file 1: Figures S4 and Figure S5). The visual juxtaposition of species phylogeny and their core pathway profiles (reflected in the figures as color changes) allows visual discernment of metabolic diversity across the tree, as well as identifying potential annotation errors when the metabolic profile of a single genome varies distinctly from all close neighbors.

### Tools for CMM comparison and analysis

We developed a web resource for exploring, comparing, and analysing our CMMs, called the Core Model Viewer (http://coremodels.mcs.anl.gov). In this tool, a list of models and links to associated genomes, media, and FBA results can be found under “Models”. Once on a model page, comprehensive tables of model reactions, compounds, gapfilled reactions, and ETC diagrams are made available. Tables of genome, media, and FBA data are organized similarly. For comparative analysis, a subset of FBA results can be selected from the models page and compared side-by-side on a heat-map (Additional file 1: Figure S6) or on metabolic pathway maps derived from KEGG [57, 58]. These tools permit the comparison of gene presence/absence, gapfilled reactions, FBA analyses, and ETC data for the selected models and enable researchers to further explore the models and results presented here.

## Abbreviations

AEA, alternate electron acceptors; BDOH, 2,3 butanediol; CMM, core metabolic models; CMT, core model template; ED pathway, Entner-Doudoroff pathway; ETC, electron transport chain; FBA, flux balance analysis; GPR, gene protein-reaction; OF, objective function; OTU, operational taxonomic units

## Additional files

Additional file 1: Supplemental figures and figure descriptions. In addition, descriptions for each supplemental data tabs in “Additional file 2” are included at the end of the document. (DOCX 17183 kb) Additional file 2: Supplemental data table associated with this study. There are twelve data tabs included in this table. Description for each data tab is at the end of “Additional file 1” document. (XLSX 2418 kb)
