# Supplementary material for: Modeling central metabolism and energy biosynthesis across microbial life
Source: BMC Genomics. 2016 Aug 8;17:568. doi: 10.1186/s12864-016-2887-8 (PMC4977884; doi:10.1186/s12864-016-2887-8)
Supplement: Additional file 1: — Supplemental figures and figure descriptions. In addition, descriptions for each supplemental data tabs in “Additional file 2” are included at the end of the document. (DOCX 17183 kb) [file 12864_2016_2887_MOESM1_ESM.docx]

Figure S1.

Core model pathway map displays major sugar degradation (glycolysis, Enter-Doudoroff, pentose phosphate), TCA cycle and fermentation pathways. Central metabolic pathway metabolites produce key precursors that eventually lead to production of cell materials required for cell growth. We have used biomass biosynthesis equation (Varma and Palsson 1993) in analyzing core metabolic model’s ability to produce these key metabolites in central metabolism and analysis on gapfill reactions for each model (Figure 4). Metabolites that are presented in the biomass equation are colored in red.


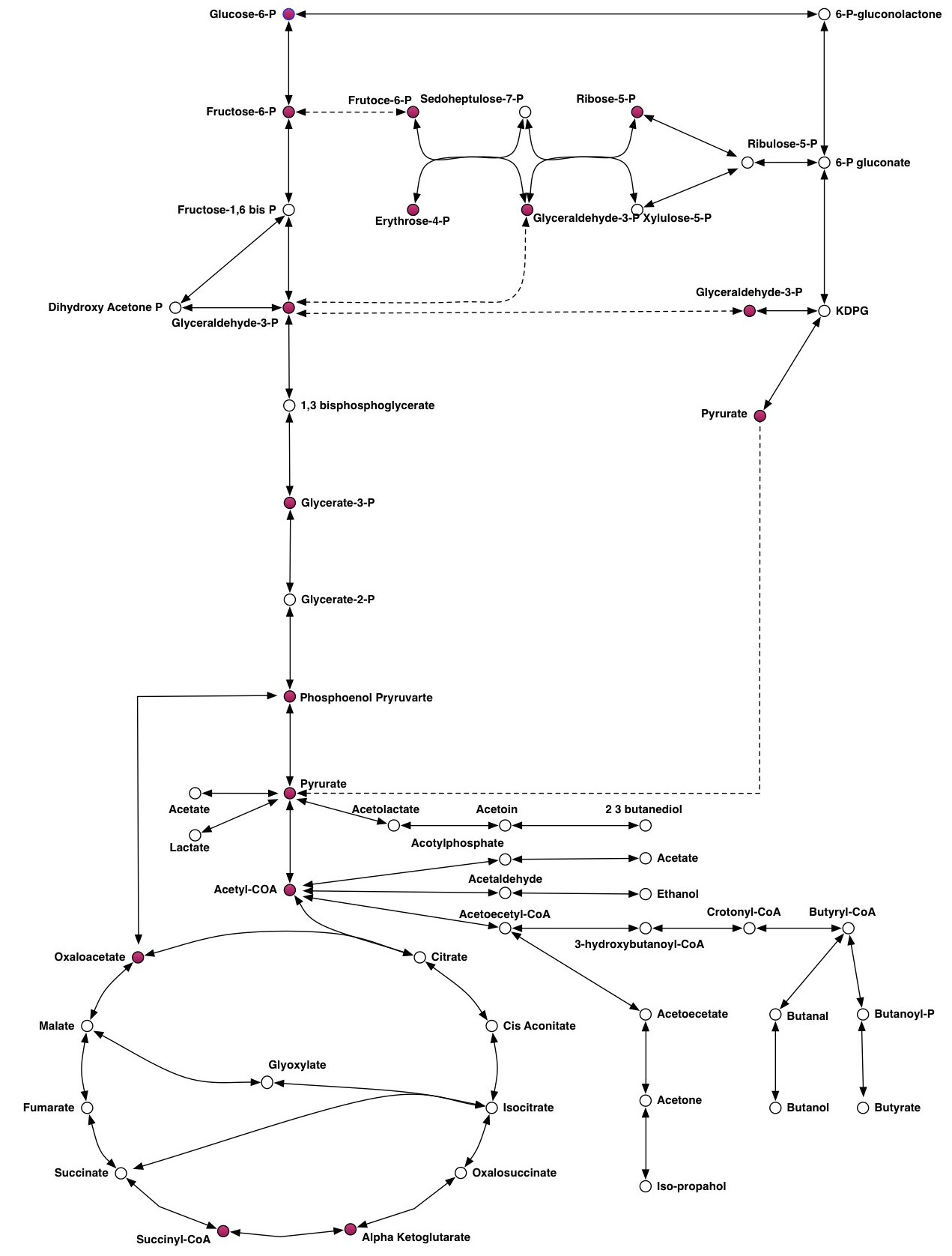


Figure S2.

Number of core reactions in core models among major phylogenetic groups

Number of core reactions in core models among major phylogenetic groups. Green bars indicate the 75th percentile and purple bars indicate the 25th percentile in the number of reactions. Error bars indicate the lowest and highest number of reactions for each phylogenetic group. The table below summarizes reaction statistics in major phylogenetic groups

| Phylogenetic Group | Approximate number of reactions | | Reaction variation | Total number of members in the group |
| --- | --- | --- | --- | --- |
| Actinobacteria | 104-129 | | 25 | 758 |
| Alphaproteobacteria | 88-142 | | 54 | 573 |
| Bacilli | 76-133 | | 57 | 1847 |
| Betaproteobacteria | 106-145 | | 39 | 385 |
| Spirochaetia | 68-96 | | 28 | 201 |
| Mollicutes | 40-52 | | 12 | 149 |
| Clostridia | 78-93 | | 15 | 389 |
| Bacteroidets | 87-105 | | 18 | 317 |
| Gammaproteobacteria | | 135-152 | 17 | 2451 |

Figure S3

Core models were analyzed for their ability to produce fermentation products (acetate, ethanol, lactate, BDOH, butyrate, butanol, acetone, formate) by finding presence of fermentation pathways and then summarized the results by their taxonomic groups.


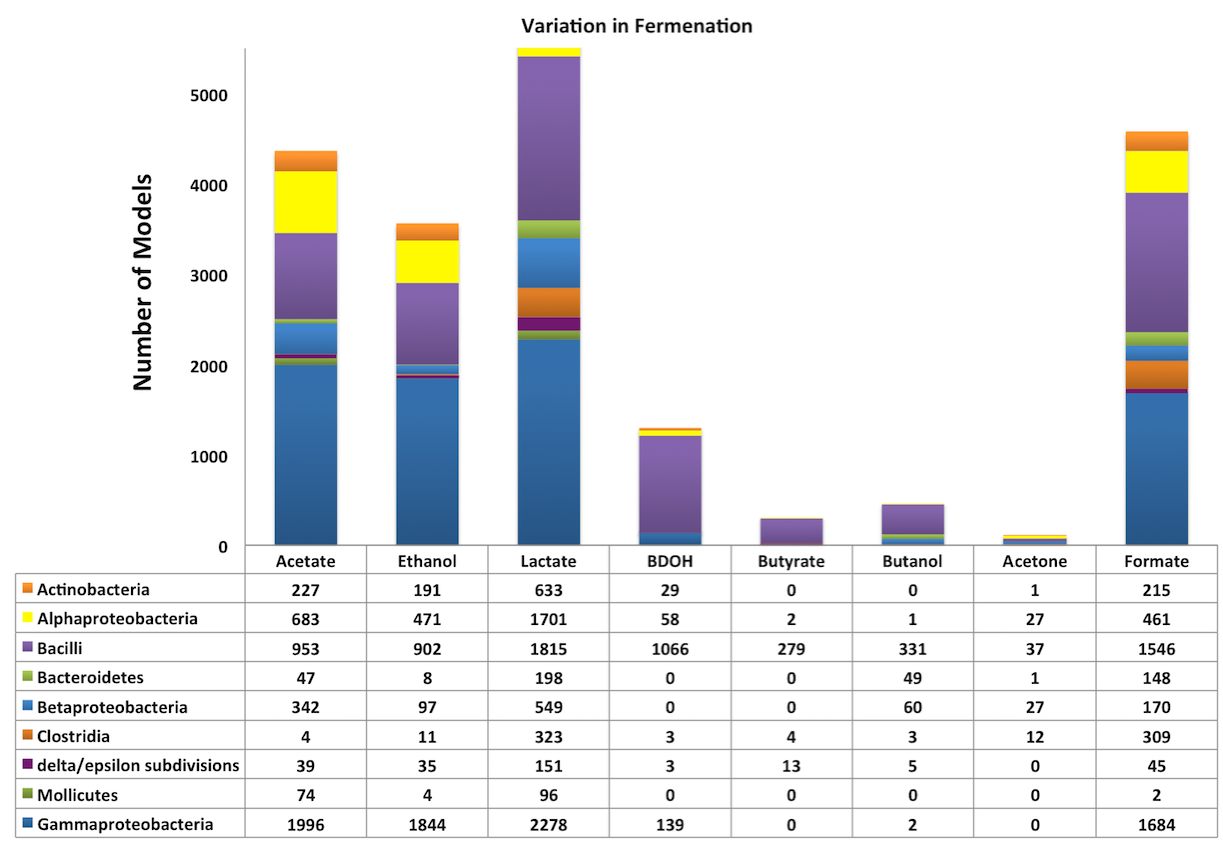


Figure S4.

Pathway conservation analysis on central metabolism

Microbial life tree (16S OTU_98.5_) depicting the presence and absence of sugar degradation pathways glycolysis, gluconeogenesis, and Enter-Doudoroff. The name of the organism and the phylum can be found at the leaf of the tree. The coloured branches depict which clades gained or lost certain metabolic pathways. The curved arrow shows the range of the group Gammaproteobacteria, and the straight arrows shows the regions where genus *Escherichia* and *Salmonella* (purple) (A), (B) Buchnera (green), (C) Shewanella (light blue) and (D)Pseudomonas (light blue) groups having different phenotypes (as explained in the text) with the same taxonomic group Gammaproteobacteria. (High-resolution image of this figure is available at <http://coremodels.mcs.anl.gov/data/supp-fig-4.pdf>)

| 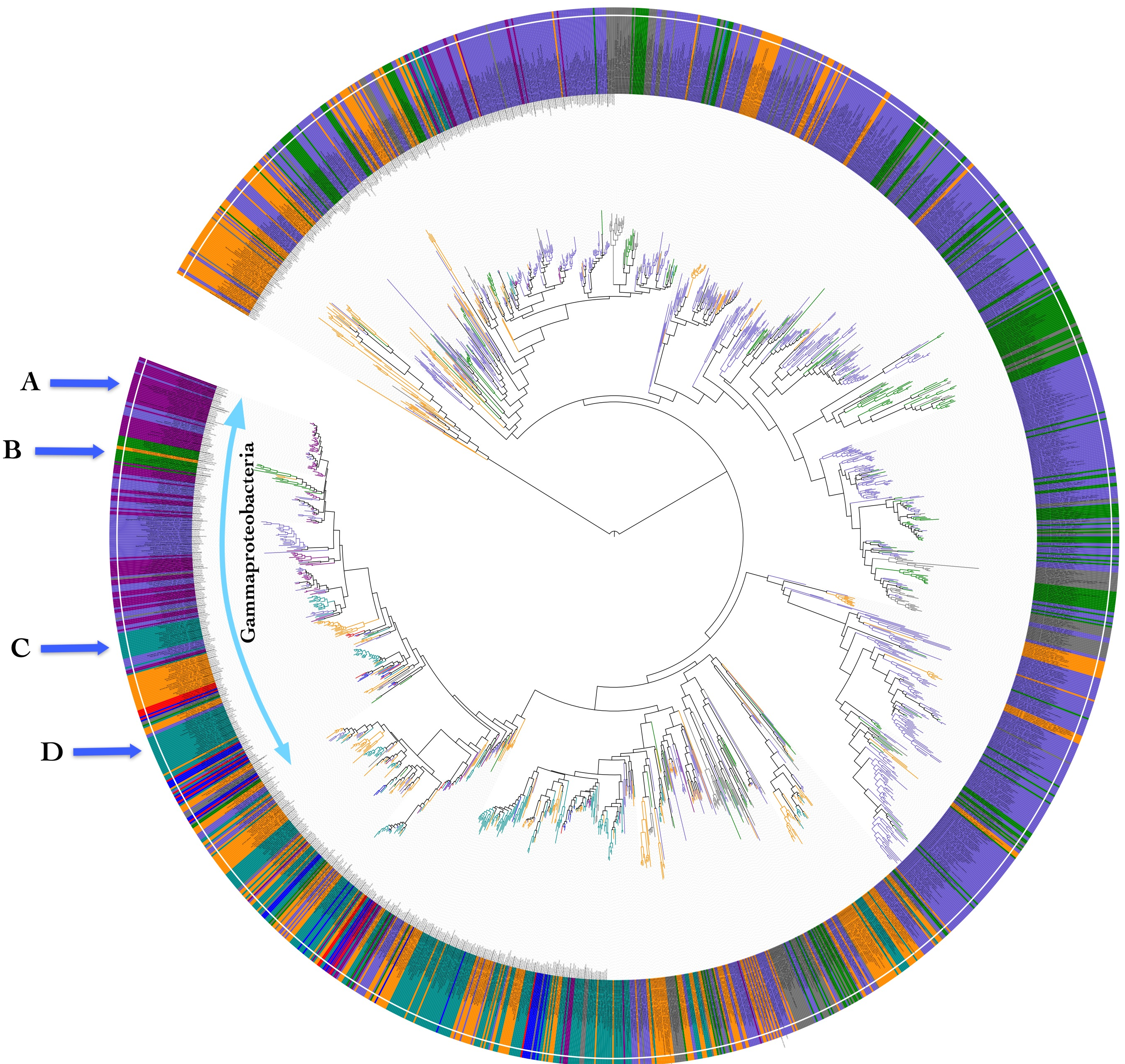 |
| --- |

Figure S5.

Variations of respiration in core metabolic models.

16S OTU_98.5_ phylogenetic tree depicting patterns in the presence of aerobic and anaerobic electron transport chains. Phylum and organism names can be found at the leaf of the tree. The coloured leafs of the tree shows the type of respiration as depicted in the color scale below. . (High-resolution image of this figure is available at <http://coremodels.mcs.anl.gov/data/supp-fig-5.pdf>)

Figure S6.

A Heat map generated using tools in core model viewer website that compare multiple FBA simulations based on core metabolic models.

Heat map shows the presence (blue) and absence (white) of each reaction for selected core metabolic models and the intensity of the fluxes (shades of red). FBA simulations performed on multiple minimal media conditions.


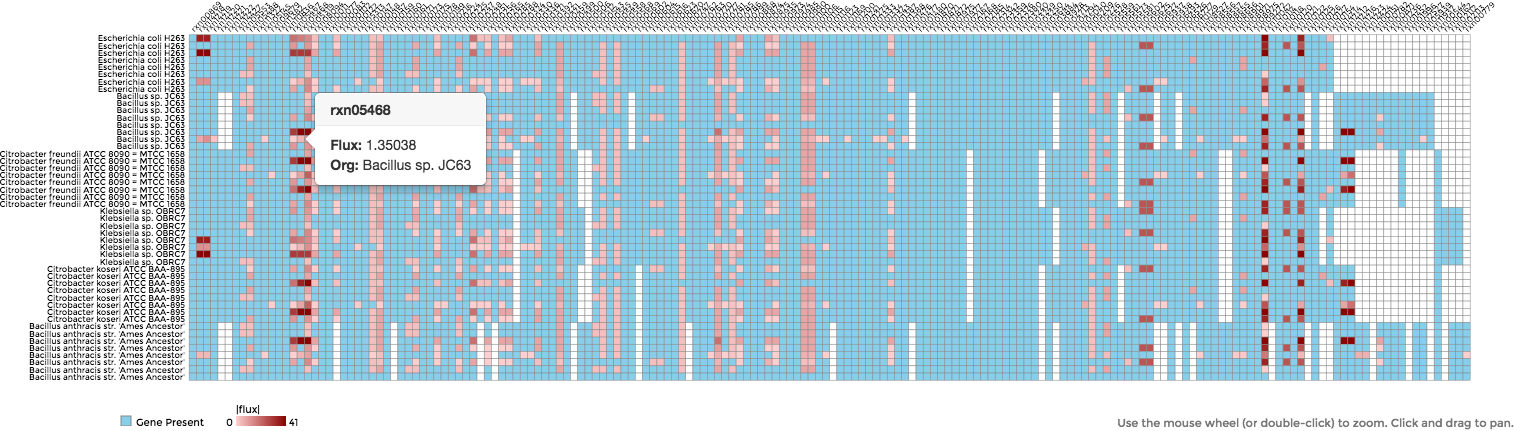


**Data Tables in Supplementary Information**

Table S1

Table 1 lists all organisms included in this study and there taxonomic classifications. The genomic content of these organisms can be viewed through SEED viewer (<http://pubseed.theseed.org/>). First column of the table displays the pubSEED genome id in reference to pubSEED genome database.

Table S2

Table 2 lists number of core models organized by their taxonomic group. For an instance there are 2451 core models belongs to the taxonomic group Gammaproteobacteria.

Table S3

Table 3 lists detail reaction information found in the core model template. Table columns display reaction id, directionality of each reaction, biochemical equation and the gene protein reaction (GPR) association information. Under GPR association information, RAST derived functional roles are listed separated by “|” sign for each reaction.

Table S4

Table 4 lists all organisms and their pathway presence (represent by 1) and absence (represent by 0) data used in this study. We have considered 12 pathways for the analysis. Organism names and their pubSEED genome ids also listed.

Table S5

Table 5 lists Flux Balance Analysis of core models. Core models with ATP biosynthesis equation simulated with seven different media conditions and collected the objective values. Data is organized by their taxonomic classification.

Table S6

Table 6 lists the genome names of the core models that are unable to produce ATP using existing RAST annotation derived reactions. These models were simulated on seven different media combinations that consist of different electron acceptors.

Table S7

Table 7 lists the gapfilling analysis data of core models with biosynthesis biomass equation (Varma, 1993 #68). Core models are listed with the total number of gapfilled reactions, total number of reactions in the model, biochemical equation of gapfilling reactions and organized by their taxonomic groups.

Table S8

Table 8 lists the genome ids and their presence and absence of aerobic and anaerobic respiration chains. If both types of respiration chains are present for a given organism then they are classified as facultative. In addition table also lists organism that does not have any type of respiration chain.

Table S9

Table 9 lists the Boolean rules that were used to determine pathway presence and absence on each model. The reaction ids are listed with mapping reaction/enzyme names.

Table S10

Table 10 lists the media formulations used in this study. Media formulations are listed by their compound ids, compound names, formula and charge.

Table S11

Table 11 lists the analysis based on core model growth on alternate carbon sources. Core models that were not able to grow using glucose as the sole carbon source, we explored the capacity of these models to utilize one or more alternative carbon sources, including glycerol, lactate, succinate and ribose.

Table S12

Table 12 lists the components and associated coefficients of the biomass objective function used in this study.

References

Varma, A. and B. O. Palsson (1993). "Metabolic capabilities of Escherichia-coli.1. Synthesis of biosynthetic precursors and cofactors." Journal of Theoretical Biology **165**(4): 477-502.
